# Alcohol’s Impact on Kidney Function

**Published:** 1997

**Authors:** Murray Epstein

**Affiliations:** Murray Epstein, M.D., is professor of medicine in the Nephrology Section at the University of Miami School of Medicine, Miami, Florida

**Keywords:** kidney function, kidney disorder, disorder of fluid or electrolyte or acid-base balance, alcoholic liver disorder, hormones, body fluid, blood circulation, blood pressure, sodium, potassium, phosphates, magnesium, calcium, literature review

## Abstract

Both acute and chronic alcohol consumption can compromise kidney function, particularly in conjunction with established liver disease. Investigators have observed alcohol-related changes in the structure and function of the kidneys and impairment in their ability to regulate the volume and composition of fluid and electrolytes in the body. Chronic alcoholic patients may experience low blood concentrations of key electrolytes as well as potentially severe alterations in the body’s acid-base balance. In addition, alcohol can disrupt the hormonal control mechanisms that govern kidney function. By promoting liver disease, chronic drinking has further detrimental effects on the kidneys, including impaired sodium and fluid handling and even acute kidney failure.

A cell’s function depends not only on receiving a continuous supply of nutrients and eliminating metabolic waste products but also on the existence of stable physical and chemical conditions in the extracellular fluid^1^ bathing it. Among the most important substances contributing to these conditions are water, sodium, potassium, calcium, and phosphate. Loss or retention of any one of these substances can influence the body’s handling of the others. In addition, hydrogen ion concentration (i.e., acid-base balance) influences cell structure and permeability as well as the rate of metabolic reactions. The amounts of these substances must be held within very narrow limits, regardless of the large variations possible in their intake or loss. The kidneys are the organs primarily responsible for regulating the amounts and concentrations of these substances in the extracellular fluid.

In addition to their role in regulating the body’s fluid composition, the kidneys produce hormones that influence a host of physiological processes, including blood pressure regulation, red blood cell production, and calcium metabolism. Besides producing hormones, the kidneys respond to the actions of regulatory hormones produced in the brain, the parathyroid glands in the neck, and the adrenal glands located atop the kidneys.

Because of the kidneys’ important and varied role in the body, impairment of their function can result in a range of disorders, from mild variations in fluid balance to acute kidney failure and death. Alcohol, one of the numerous factors that can compromise kidney function, can interfere with kidney function directly, through acute or chronic consumption, or indirectly, as a consequence of liver disease. This article first reviews direct effects of alcohol on kidney structure, function, and regulation, highlighting relevant effects associated with liver disease. Following this discussion, the article takes a more in-depth look at several important indirect effects of alcohol on the kidneys that occur once liver disease has become established.

## Gross and Microscopic Changes

One way in which alcohol directly affects the kidneys is by altering the form and structure of this pair of organs, as demonstrated by various animal studies. For example, in an early study on dogs (Chaikoff et al. 1948), investigators observed several striking alterations after chronic alcohol administration. The basement membrane of the glomerulus (see sidebar figure) became abnormally thickened and was characterized by cell proliferation. Further changes included enlarged and altered cells in the kidney tubules. In another study, Van Thiel and colleagues (1977) compared kidney structure and function in alcohol-fed and control rats. The alcohol-fed group experienced kidney swelling and significantly reduced kidney function; in addition, under microscopic examination, the kidneys of alcohol-fed rats were found to have cells enlarged with increased amounts of protein, fat, and water, compared with those of the control animals.

**Kidney Structure and Function**

Cushioned in fatty tissue near the base of the spinal column, the kidneys are efficiently designed organs that perform two primary tasks in the body: excretion of metabolic end products and precise regulation of body fluid constituents. As they accomplish these tasks, the kidneys form and collect urine, which exits through the ureters to the bladder.

Each of the 2 million functional units (i.e., nephrons) in a pair of normal kidneys forms urine as it filters blood plasma of substances not needed by the body. Within each nephron, blood plasma enters a tiny ball of unusually permeable capillaries (i.e., the glomerulus), filters into a capsule that surrounds the glomerulus, then flows through a long, looping conduit called the nephron tubule.

**Figure:**
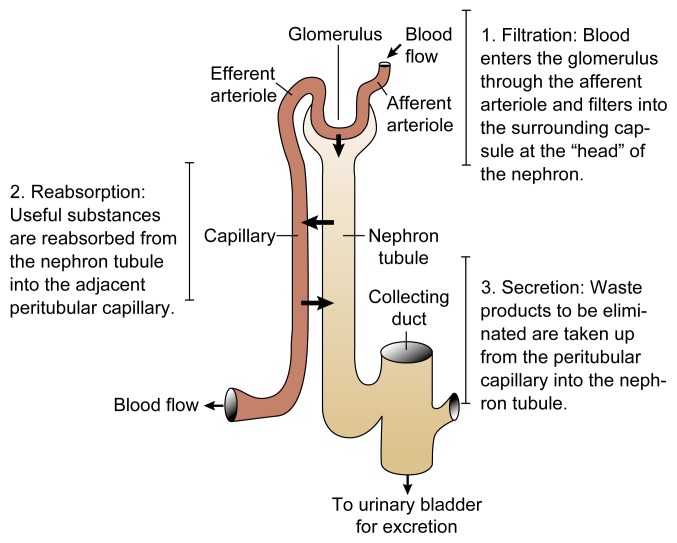
Urine formation. Three basic processes—glomerular filtration, tubular reabsorption, and tubular secretion—contribute to urine formation, as shown in this schematic.

As the plasma filtrate passes along this channel, the substances the body needs to conserve are reabsorbed into an extensive network of capillaries that wrap the nephron tubule. Many electrolytes and 99 percent of the water in the filtrate are reabsorbed, while waste products, such as urea (an end product of protein metabolism formed chiefly in the liver) and creatinine (an end product of muscle metabolism), as well as excess electrolytes (particularly sodium, potassium, chloride, and hydrogen ions), continue to journey along the tubule. Small amounts of unwanted substances also are secreted directly into the nephron tubules. Together, the filtered and secreted substances form urine (see figure) and eventually trickle into a series of progressively larger collecting ducts. Each 4.5-inch-long kidney contains about 250 of the largest collecting ducts, each duct transmitting urine from approximately 4,000 nephrons.

Of the 48 gallons of filtrate processed through the nephrons of the kidneys each day, only about 1 to 1.5 quarts exit as urine. During this filtering process, substances are reabsorbed or secreted to varying degrees as the filtrate passes through the distinct segments of the nephron tubule.

***Regulating Electrolyte Levels and Fluid Volume***

The kidney tubules play an important role in keeping the body’s water and electrolyte levels in equilibrium. In many cases, control mechanisms govern the rate of reabsorption or secretion in response to the body’s fluctuating needs (see table for a summary of the body processes influenced by key electrolytes). Under the influence of antidiuretic hormone (ADH), for example, the tubules can create either a concentrated urine, to discharge excess solutes and conserve water, or a dilute urine, to remove extra water from body fluids. In the first case, when body fluids become too concentrated with solutes, the pituitary gland produces abundant ADH, which induces the kidneys to conserve water and concentrate urine, an important ability that enables thriving in an environment where water may be scarce. In the absence of ADH, when body fluids are overly dilute, the kidneys dilute the urine, allowing more water to leave the body. “Normal” urine flow rate is 1 milliliter per minute (i.e., approximately 1 to 1.5 L/day), but this rate can vary widely, depending on water intake or dehydration level, for instance.

**Table:** Key Electrolytes in the Body

Ion	Major Physiological Roles
Sodium (Na^+^)	Primary positive ion in extracellular fluid. Together with potassium, maintains electrolyte balance across all cell membranes. Vital to many basic physiological functions.
Potassium (K^+^)	Primary positive ion in intracellular fluid. Together with sodium, maintains electrolyte balance across all cell membranes. Vital to many basic physiological functions.
Magnesium (Mg^2+^)	Required for activity of many enzymes.
Calcium (Ca^2+^)	Major mineral in body and component of bone. Helps maintain normal heartbeat and nerve and muscle function.
Chloride (Cl^−^)	Primary negative ion in extracellular fluid. Plays a role in nerve function and other metabolic processes.
Phosphate (HPO_4_^2^^−^)	Primary negative ion in intracellular fluid. Serves as an important buffer to maintain proper pH. Phosphorus is a major component of bone and is involved in almost all metabolic processes.

The kidneys continuously perform their tasks of purifying and balancing the constituents of the body’s fluids. Although resilient, the kidneys can deteriorate as a result of malnutrition, alcohol abuse or dependence, or liver and other diseases. Healthy kidneys are vital to the function of all the body’s organs and systems.

— Mary Beth de Ribeaux

BibliographyGuytonACHuman Physiology and Mechanisms of Disease5th edPhiladelphiaW.B. Saunders1992195246RhoadesRPflanzerRHuman Physiology2d edFort WorthSaunders College Publishing1992823909

*Mary Beth de Ribeaux is a science editor of* Alcohol Health & Research World

Similarly, clinicians long have noted significant kidney enlargement (i.e., nephromegaly) in direct proportion to liver enlargement among chronic alcoholic^2^ patients afflicted with liver cirrhosis. Laube and colleagues (1967) suggested that both cellular enlargement and cell proliferation contribute to such nephromegaly. In alcoholic patients with cirrhosis, these investigators reported a 33-percent increase in kidney weight, whereas they observed no appreciable kidney enlargement in alcoholic patients without cirrhosis compared with control subjects (Laube et al. 1967).

## Blood-Flow Changes

Normally the rate of blood flow, or perfusion, (i.e., hemodynamics) through the kidneys is tightly controlled, so that plasma can be filtered and substances the body needs can be reabsorbed under optimal circumstances (see sidebar). Established liver disease impairs this important balancing act, however, by either greatly augmenting or reducing the rates of plasma flow and filtration through the glomerulus. Investigators have not yet fully explained the mechanisms underlying this wide range of abnormalities, though, and have devoted little attention to alcohol’s effects on kidney hemodynamics in people who do not have liver disease.

The few studies focusing on alcohol’s direct effects on perfusion in human kidneys suggest that regulatory mechanisms retain control over this component of kidney function despite alcohol consumption. Even at high blood alcohol levels, only minor fluctuations were found in the rates of plasma flow and filtration through the kidneys (Rubini et al. 1955). Additional studies are needed to confirm these observations, however.

Results of subsequent studies in animal models seem to vary according to the species examined, the route and dose of alcohol administration, and the length of time after administration for which the study groups were observed. For example, some studies implied that acute alcohol consumption does not significantly change kidney hemodynamics or sodium excretion in dogs, but these studies did not extend beyond 6 hours after alcohol ingestion. In contrast, earlier studies that examined dogs for a longer period reported that a single dose of 3 grams of alcohol per kilogram of body weight (g/kg) elevated plasma volume between 10 and 26 hours following alcohol ingestion (Nicholson and Taylor 1940).^3^

Another study with dogs (Beard et al. 1965) disclosed that the effects of chronic alcohol consumption endured even longer. The investigators noted increased plasma and extracellular fluid volume 1 week after chronic alcohol ingestion, and these volume expansions persisted for the remaining 7 weeks of the study. Similar alterations have been found in body fluid volumes among chronic alcoholic patients.

## Effects on Fluid and Electrolyte Balance

One of the main functions of the kidneys is to regulate both the volume and the composition of body fluid, including electrically charged particles (i.e., ions), such as sodium, potassium, and chloride ions (i.e., electrolytes). However, alcohol’s ability to increase urine volume (i.e., its diuretic effect) alters the body’s fluid level (i.e., hydration state) and produces disturbances in electrolyte concentrations. These effects vary depending on factors such as the amount and duration of drinking, the presence of other diseases, and the drinker’s nutritional status (see table, p. 90).

### Fluid

Alcohol can produce urine flow within 20 minutes of consumption; as a result of urinary fluid losses, the concentration of electrolytes in blood serum increases. These changes can be profound in chronic alcoholic patients, who may demonstrate clinical evidence of dehydration.

As most investigators now agree, increased urine flow results from alcohol’s acute inhibition of the release of antidiuretic hormone (ADH), a hormone also known as vasopressin, which normally promotes the formation of concentrated urine by inducing the kidneys to conserve fluids. In the absence of ADH, segments of the kidney’s tubule system become impermeable to water, thus preventing it from being reabsorbed into the body. Under these conditions, the urine formed is dilute and electrolyte concentration in the blood simultaneously rises. Although increased serum electrolyte concentration normally activates secretion of ADH so that fluid balance can be restored, a rising blood alcohol level disrupts this regulatory response by suppressing ADH secretion into the blood.

Interestingly, age makes a difference in how rapidly the body escapes alcohol’s ADH-suppressive effect. People older than age 50 overcome suppression of ADH more quickly than their younger counterparts do, despite reaching similar serum electrolyte concentrations after alcohol consumption. In older people, ADH levels sharply increase following alcohol intake, perhaps in part because sensitivity to increased electrolyte concentration is enhanced with age. It is not known whether chronic alcoholic patients experience a similar difference in the ADH response as they age, however.

### Sodium

The serum sodium level is determined by the balance of fluid in relation to that of sodium: Not enough fluid in the body results in a sodium concentration that is too high (i.e., hypernatremia), whereas excessive amounts of fluid produce a sodium concentration that is too low (i.e., hyponatremia). Hyponatremia does not constitute merely a biochemical abnormality but most likely has clinical consequences as well (e.g., impaired mental activity, neurological symptoms, and, in extreme instances, seizures).

“Beer drinkers’ hyponatremia” is a syndrome that appears to result from an intake of excessive fluid in the form of beer. Hilden and Svendsen (1975) observed hyponatremia in five patients who drank at least 5 liters of beer per day (L/d) without any other nourishment. (For comparison, a person’s normal fluid intake averages a little more than 2 L/d.) Because beer contains few dissolved substances (i.e., solutes), such as sodium, these patients apparently lacked a sufficient quantity of solutes to stimulate the kidneys to eliminate excess fluid.

Although fluid overload—not alcohol itself—is considered the major contributor to beer drinkers’ hyponatremia, alcohol does appear to directly influence the kidney’s handling of sodium and other electrolytes, potentially resulting in hypernatremia. In a study by Rubini and colleagues (1955), subjects who consistently drank about 4 ounces (oz) of 100-proof bourbon whiskey experienced decreased sodium, potassium, and chloride excretion (i.e., increased retention of solutes). Although some exceptions exist, several historical studies have reported similar modest reductions in sodium and potassium excretion following alcohol use.

In general, however, neither acute nor chronic alcohol consumption directly causes significant changes in serum sodium concentrations, although impaired sodium excretion is a frequent complication of advanced liver disease, as discussed later in this article.

### Potassium

Normally the kidneys are a major route of potassium ion excretion and serve as an important site of potassium regulation. Alcohol consumption historically has been found to reduce the amount of potassium excreted by the kidneys (e.g., Rubini et al. 1955), although the body’s hydration state may help determine whether potassium excretion will increase or decrease in response to alcohol. Levels of potassium, like those of sodium, also can affect the way the kidneys handle fluid elimination or retention. In addition, potassium depletion has been proposed to exacerbate hyponatremia through any of several mechanisms (Epstein 1992): For example, potassium losses may stimulate ADH activity, thereby increasing the amount of fluid reabsorbed and causing the body’s sodium concentration to decrease as a result. Alternatively, potassium losses may increase thirst, also through hormonal mechanisms, thereby promoting increased fluid intake.

### Phosphate

Low blood levels of phosphate commonly occur acutely in hospitalized alcoholic patients, appearing in more than one-half of severe alcoholism cases. Indeed, when the condition does not appear, clinicians treating alcoholic patients should suspect that another problem is masking the recognition of low phosphate levels, such as ongoing muscle dissolution, excess blood acidity (i.e., acidosis), inadequate blood volume, or kidney failure.

Several mechanisms may contribute to abnormally low phosphate levels (i.e., hypophosphatemia) (see box). Simply lacking an adequate amount of phosphate in the diet is one possible reason for phosphate deficiency. For severely alcoholic patients who eat poorly, such a nutritional deficit may be an important contributor to hypophosphatemia.

**Causes of Low Phosphate Levels in Alcoholics**

The following causes may underlie low phosphate levels in severe alcoholics:

- Phosphorus deficiency in the diet
- Increased blood pH due to prolonged rapid breathing
- Insulin administration
- Administration of nutrients beyond normal requirements (in hospital settings)
- Excessive excretion in urine
- Magnesium deficiency.

SOURCE: Adapted from Epstein, M. Alcohol and the kidney. In: Lieber, C.S., ed. *Medical and Nutritional Complications of Alcoholism: Mechanisms and Management*. New York: Plenum Medical Book Company, 1992. p. 498.

Another potential cause of hypophosphatemia in alcoholic patients is hyperventilation, which can occur during alcohol withdrawal. Prolonged rapid, shallow breathing results in excessive loss of carbon dioxide and decreased blood acidity (i.e., alkalosis), which in turn activates an enzyme that enhances glucose breakdown. In glucose breakdown, phosphate becomes incorporated into various metabolic compounds, ultimately lowering blood levels of phosphate. As the rate of glucose breakdown increases, profound hypophosphatemia potentially can result.

Insulin administration also can lead to mild hypophosphatemia, because it decreases cellular acidity. Although insulin more likely plays a contributory, rather than principal, role in producing hypophosphatemia in alcoholic patients, there are clinical implications to consider. Both glucose and amino acids are powerful triggers for insulin release, and hospitalized alcoholic patients frequently receive intravenous fluids containing these nutrients. Physicians thus should be prepared to respond if hypophosphatemia develops. A similar concern applies to another treatment that may lead to hypophosphatemia: overfeeding patients beyond normal nutrient requirements in an attempt to replace dietary deficiencies.

Alcoholic patients also may develop low blood levels of phosphate by excreting too much of this ion into their urine. Typically, chronic alcoholic patients are losing up to 1.5 g/d of phosphate through their urine when they have reached the point of being sick enough to accept hospitalization. (For comparison, a normal healthy person excretes 0.7 to 0.8 g/d.) Over the next several days of hospitalization, these patients often excrete virtually no phosphate in their urine; simultaneously, their blood phosphate levels dip to low levels before returning to normal. The combination of low phosphate excretion and low blood levels indicates that phosphate is simply being shifted from the bloodstream into body cells, implying that kidney dysfunction is not a likely cause of phosphate wasting in this case.

Alcohol can induce abnormally high phosphate levels (i.e., hyperphosphatemia) as well as abnormally low levels. In fact, hyperphosphatemia often precedes hypophosphatemia. Alcohol consumption apparently leads to excessive phosphate levels by altering muscle cell integrity and causing the muscle cells to release phosphate. This transfer of phosphate out of muscle cells and into the bloodstream results in an increased amount of phosphate passing through the kidneys’ filtering system. In response, reabsorption of phosphate diminishes and excretion in urine increases in an effort to return blood levels of this ion to normal.

### Magnesium

Chronic alcoholism is the leading cause of low blood levels of magnesium (i.e., hypomagnesemia) in the United States (Epstein 1992). Often it occurs simultaneously with phosphate deficiencies, also frequently encountered among alcoholic patients. Hypomagnesemia responds readily to magnesium supplementation treatment, however.

Several alcohol-related mechanisms can result in hypomagnesemia. Studies historically have shown that alcohol consumption markedly increases magnesium excretion in the urine and may affect magnesium levels in other ways as well. For example, when rats are given alcohol, they also require significant magnesium in their diets, suggesting that alcohol disrupts absorption of this nutrient from the gut. Investigators have speculated that alcohol or an intermediate metabolite directly affects magnesium exchange in the kidney tubules (Epstein 1992).

### Calcium

Early studies showed that alcohol consumption markedly increases calcium loss in urine. In severely ill alcoholic patients, low blood levels of calcium occur about as often as low blood levels of phosphate and can cause convulsions or potentially life-threatening muscle spasms when respiratory muscles are involved. Alcoholic patients with liver disease often have abnormally low levels of a calcium-binding protein, albumin, and also may have impaired vitamin D metabolism; either of these two factors could result in reduced blood levels of calcium (i.e., hypocalcemia). Muscle breakdown and magnesium deficiency are other potential causes of hypocalcemia in alcoholic patients. A direct effect of alcohol in reducing calcium levels is suggested by at least one experimental study: Dogs became hypocalcemic after administration of alcohol above a critical threshold amount of approximately 1 g/kg (Money et al. 1989).

## Body Fluid Volume and Blood Pressure

Chronic alcohol consumption may cause both fluid and solutes to accumulate, thereby increasing the overall volume of body fluids. In turn, such expansion of body fluid volume can contribute to high blood pressure, a condition often seen among chronic alcoholic patients.

The association between increased blood pressure and alcohol consumption has been recognized at least since 1915, when Lian reported the prevalence of high blood pressure (i.e., hypertension) in relation to the drinking habits of French army officers. More recent studies have substantiated this link. For example, in the large-scale Kaiser-Permanente study, in which blood pressure measurements and alcohol histories were obtained from more than 80,000 men and women, the association between blood pressure and drinking was found to be independent of age, sex, ethnicity, weight, smoking habit, and social class (Klatsky et al. 1977).

Clinical studies of hypertensive patients have demonstrated that reducing alcohol intake lowers blood pressure and resuming consumption raises it. Although the mechanisms responsible for these effects have not been established, an experimental study by Chan and Sutter (1983) offers some insight. In this study, male rats given 20-percent alcohol in their drinking water for 4 weeks experienced decreased urinary volume and sodium excretion as well as increased blood concentrations of hormones that raise blood pressure by constricting blood vessels. The results of this study suggest that alcohol’s influence on blood pressure may be attributable, at least in part, to its effects on the production of hormones that act on the kidneys to regulate fluid balance or that act on blood vessels to constrict them.

## Acid-Base Balance Effects

Most of the metabolic reactions essential to life are highly sensitive to the acidity (i.e., hydrogen ion concentration) of the surrounding fluid. The kidneys play an important role in regulating acidity, thereby helping determine the rate at which metabolic reactions proceed. Alcohol can hamper the regulation of acidity, thus affecting the body’s metabolic balance.

One example of an alcohol-related acid-base disturbance already has been mentioned in relation to low levels of phosphate (i.e., respiratory alkalosis resulting from hyperventilation during alcohol withdrawal). Other acid-base disturbances are possible as a result of excessive alcohol consumption. These disturbances increase the kidneys’ workload in restoring acid-base balance through formation of an acidic or basic (i.e., alkaline) urine. For instance, the opposite of respiratory alkalosis can occur when a person becomes extremely intoxicated. Because alcohol is a central nervous system depressant, it may slow the rate of breathing as well as reduce the brain’s respiratory center’s sensitivity to carbon dioxide levels. As a result, excess carbon dioxide accumulates, and the body’s acid level subsequently increases. Respiratory acidosis is rare but carries an ominous prognosis when it occurs.

Excess blood acidity in alcoholic patients more often results from severe elevations of a product of glucose metabolism (i.e., lactate), which can be induced by alcohol consumption as well as other factors. A potentially serious condition known as alcoholic ketoacidosis is another disorder associated with abnormally high blood acidity. Characterized by an abnormal accumulation of ketone bodies, which are substances manufactured in the liver and used as reserve fuels for muscle and brain tissue, ketoacidosis also is a complication of uncontrolled diabetes and starvation. Typically, alcoholic ketoacidosis occurs in chronic alcohol abusers following a severe binge in which they consume alcoholic beverages and nothing else over several days. Certain people appear to be particularly prone to alcoholic ketoacidosis and may develop the condition repeatedly, but the reason for their susceptibility remains unknown (Epstein 1992).

Additional causes of nonrespiratory acidosis include drinking nonbeverage alcohol (e.g., antifreeze or wood alcohol), which alcoholics sometimes resort to consuming when beverage alcohol is unavailable; aspirin overdose; and administration of paraldehyde, a sedative used for alcohol withdrawal.

Despite the multiple possible causes of acidosis, disturbances in acid-base balance are more frequently manifested as low acidity (i.e., alkalosis). Alkalosis was present in 71 percent of patients with established liver disease in 11 studies, and respiratory alkalosis was the most common disturbance in 7 of the studies (Oster and Perez 1996). If an acute alcoholic binge induces extensive vomiting, potentially severe alkalosis may result from losses of fluid, salt, and stomach acid.

Like the kidneys, the liver plays an important role in maintaining acid-base balance. Liver diseases—including alcohol-induced liver problems—disrupt this function and can contribute directly or indirectly to a wide range of acid-base disturbances.

## Regulatory Effects

To keep the kidneys functioning optimally and to maintain functional stability (i.e., homeostasis) in the body, a variety of regulatory mechanisms exert their influence. Alcohol can perturb these controls, however, to a degree that varies with the amount of alcohol consumed and the particular mechanism’s sensitivity.

As an example, Puddey and colleagues (1985) evaluated the effects of hormones that regulate kidney function. Their results show not only how alcohol disrupts homeostasis but also how the body reacts to restore it. Following moderate alcohol consumption—about 24 oz—of nonalcoholic beer with 1 milliliter of alcohol per kilogram of body weight added, the investigators noted several effects. Alcohol-induced urination reduced the subjects’ plasma volume, resulting in an increased concentration of plasma sodium. In addition, the subjects’ blood pressure and plasma potassium concentration decreased. These changes in fluid volume, electrolyte balance, and blood pressure may have stimulated the activity of hormones to return body fluid volume and composition back to normal, which occurred soon after consumption.

Alcohol consumption also is known to induce a state of low blood sugar (i.e., hypoglycemia) and activate the portion of the nervous system that coordinates the body’s response to stress (i.e., the sympathetic nervous system). Both of these factors affect hormones that regulate kidney function, just as changes in fluid volume and electrolyte balance do.

## Indirect Effects

Physicians have recognized an interrelationship between kidney and liver disorders at least since the time of Hippocrates. Although a disorder in one organ can complicate a primary problem in the other (or a pathological process may involve both organs directly), kidney dysfunction complicating a primary disorder of the liver (e.g., cirrhosis) is the most clinically significant scenario. Frequently, such kidney dysfunction results from liver problems related to alcohol. In fact, most patients in the United States diagnosed with both liver disease and associated kidney dysfunction are alcohol dependent (Epstein 1992). (See the article by Maher, pp. 5–12.) Three of the most prominent kidney function disturbances that arise in the presence of established liver disease are impaired sodium handling, impaired fluid handling, and acute kidney failure unexplained by other causes (i.e., hepatorenal syndrome).

### Impaired Sodium Handling

Patients with alcohol-induced liver cirrhosis show a great tendency to retain salt (i.e., sodium chloride), and their urine frequently is virtually free of sodium. A progressive accumulation of extracellular fluid results, and this excess fluid is sequestered primarily in the abdominal region, where it manifests as marked swelling (i.e., ascites) (see figure). In addition, excess fluid accumulates in spaces between cells, clinically manifested as swelling (i.e., edema) of the lower back and legs. As long as cirrhotic patients remain unable to excrete sodium, they will continue to retain the sodium they consume in their diet. Consequently, they will develop increasing ascites and edema and experience weight gain. In some cases, vast amounts of abdominal fluid may collect, occasionally more than 7 gallons (Epstein 1996).

Rigorously limiting sodium intake, which is the first step in treating ascites, will halt fluid retention. Such sodium restriction alone may bring about a spontaneous increase in urine flow and relieve ascites, but how often this response occurs and in which patients is unknown and unpredictable. Other treatment options include various diuretic agents and, when ascites stubbornly persists, aspiration of the excess abdominal fluid. Many sodium-retaining patients who receive large quantities of sodium-free water can excrete copious amounts of dilute urine, thus demonstrating that the main kidney dysfunction associated with their ascites is an impaired ability to excrete sodium, not water.

The events leading to abnormal sodium handling in patients with cirrhosis are complex and controversial, however. Investigators have advanced several theories suggesting the involvement of a constellation of hormonal, neural, and hemodynamic mechanisms (Epstein 1996; Laffi et al. 1996). The traditional hypothesis holds that the kidneys of cirrhotic patients retain sodium in response to ascites that develops when liver dysfunction causes blood vessels to expand beyond available plasma volume (i.e., the “underfill” theory). In contrast, the “overflow” theory postulates that ascites follows when the kidneys retain sodium in response to signals sent by a dysfunctional liver to expand plasma volume. The answer to this version of the “chicken-and-egg” question remains to be elucidated.

**Figure:**
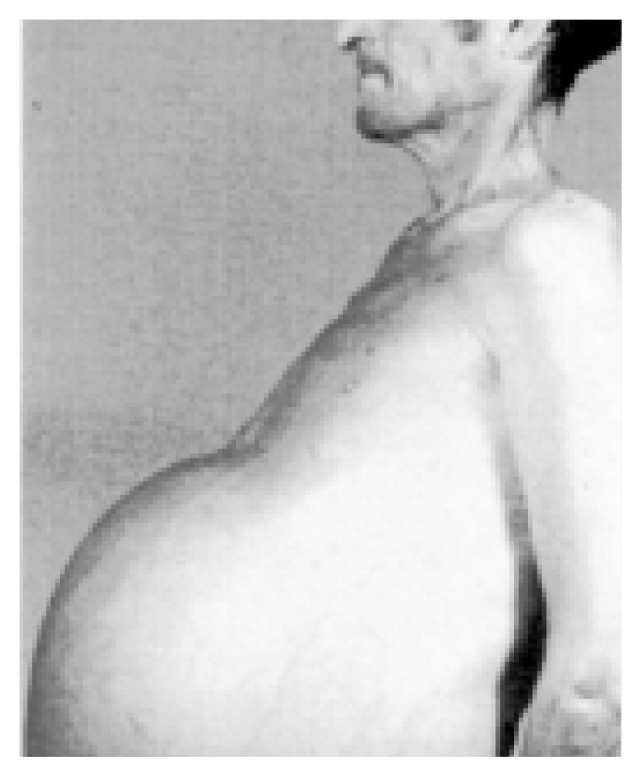
Patient with marked ascites. SOURCE*:* Reprinted with permission from Epstein, M.: *The Kidney in Liver Disease*, 4th ed. Philadelphia, PA: Hanley & Belfus, 1996.

### Impaired Fluid Handling

In many patients with liver cirrhosis, the kidneys’ ability to create dilute urine is compromised, leading to a state of abnormally low sodium concentration (i.e., hyponatremia). In hyponatremic patients, the amount of fluid retained by the kidneys is disproportionately greater than the amount of sodium retained. In other words, the kidneys’ ability to excrete excess fluid by way of dilute urine is impaired, and too much fluid is reabsorbed. Hyponatremia probably is the single most common electrolyte disturbance encountered in the management of patients with cirrhosis of the liver (Vaamonde 1996). This abnormality may reflect the severity of liver disease, but the available data do not allow correlation of kidney impairment with the degree of clinical signs of liver disease, such as ascites or jaundice.

A compromised diluting ability has important implications for the management of patients with advanced liver disease. Restricting the fluid intake of hyponatremic patients eventually should restore a normal fluid balance; unfortunately, this restriction may be difficult to implement. Patients frequently fail to comply with their physician’s orders to limit their fluid intake. Furthermore, clinicians sometimes overlook the fact that fluids taken with medications also must be restricted for these patients and mistakenly bring pitchers of juice or water to their bedsides.

The difficulties in successfully managing dilutional hyponatremia have resulted in the recent emergence of a promising class of new drugs to treat this abnormality. Specifically, drugs known as arginine vasopressin antagonists are being developed to inhibit ADH at the cell receptor level. These new drugs should dramatically facilitate treatment of cirrhotic patients with impaired fluid handling.

### Hepatorenal Syndrome

Hepatorenal syndrome may appear in patients afflicted with any severe liver disease, but in the United States, studies most often have identified alcoholic cirrhosis as the underlying disorder. Major clinical features of hepatorenal syndrome include a marked decrease in urine flow, almost no sodium excretion and, usually, hyponatremia and ascites. Blood urea nitrogen (BUN) levels and serum concentrations of the waste product creatinine are somewhat elevated, but rarely to the degree seen in patients with end-stage kidney failure when kidney disease is the primary disorder. Judgments based on such relatively modest BUN and serum creatinine increases often underestimate kidney dysfunction in patients with hepatorenal syndrome, however, because malnourished cirrhotic patients tend to have low levels of urea and creatinine.

Although hepatorenal syndrome often ensues after an event that reduces blood volume (e.g., gastrointestinal bleeding), it also can occur without any apparent precipitating factor. Some observers have noted that patients with cirrhosis frequently develop hepatorenal syndrome following hospital admission, possibly indicating that a hospital-related event can trigger the syndrome. Regardless of the precipitating factor, patients who develop kidney failure in the course of alcoholic cirrhosis have a grave prognosis.

Substantial evidence exists to support the concept that kidney failure in hepatorenal syndrome is not related to structural damage and is instead functional in nature. For example, almost 30 years ago, Koppel and colleagues (1969) demonstrated that kidneys transplanted from patients with hepatorenal syndrome are capable of resuming normal function in recipients without liver disease. In addition, Iwatsuki and colleagues (1973) and Gonwa and Wilkinson (1996) documented the return of normal kidney function in hepatorenal syndrome patients who receive liver transplants.

**Table:** How Alcoholism Contributes to Electrolyte Disturbances

Disturbance	Major Cause(s)
Low sodium level (i.e., hyponatremia)	Massive intake of solute-free fluid (e.g., beer)
Low potassium level (i.e., hypokalemia)	Dietary deficiency or gastrointestinal losses Leaky membranes Extracellular-to-intracellular shifts
Low phosphorus level (i.e., hypophosphatemia)	Dietary deficiency or malabsorption Increased cellular uptake
Low magnesium level (i.e., hypomagnesemia)	Dietary deficiency or malabsorption Phosphorus deficiency

SOURCE: Adapted from Epstein, M. Alcohol and the kidney. In: Lieber, C.S., ed. *Medical and Nutritional Complications of Alcoholism: Mechanisms and Management*. New York: Plenum Medical Book Company, 1992. p. 502.

Indeed, liver transplantation is one of two options available today for treating hepatorenal syndrome. Gonwa and Wilkinson (1996) reported a 4-year survival rate of 60 percent in hepatorenal syndrome patients who received a liver transplant, which constitutes a major step forward, considering the previous uniformly fatal course of the disease.

Another current treatment option is known as transjugular intrahepatic portosystemic shunt (TIPS), in which a bypass (i.e., shunt) between two veins inside the liver (i.e., the hepatic vein and the portal vein) is created by way of the jugular vein. Obstructions in the liver of cirrhotic patients increase pressure in the portal vein, and this effect is thought to contribute to many kidney complications. The TIPS technique was developed as a means to reduce pressure in the portal circuit and offers several advantages: Because the shunt can be inserted under local anesthesia, TIPS avoids postoperative complications associated with surgery. In addition, TIPS does not alter the anatomy of the blood vessels outside the liver, an important consideration for potential liver-transplant candidates. The less invasive nature of TIPS makes it an attractive option for treating hepatorenal syndrome, and preliminary results show that the procedure is effective (Somberg 1996). Currently, a clinical trial sponsored by the National Institutes of Health is investigating the effects of TIPS on the treatment of ascites and improvement of kidney function.

## Conclusion

Excessive alcohol consumption can have profound negative effects on the kidneys and their function in maintaining the body’s fluid, electrolyte, and acid-base balance, leaving alcoholic people vulnerable to a host of kidney-related health problems. Despite the clinical importance of alcohol’s effects on the kidney, however, relatively few recent studies have been conducted to characterize them or elucidate their pathophysiology. It is hoped that future investigations will focus on this important subject area.

## Footnotes

1 For a definition of this and other technical terms used in this article, see the glossary, pp. 93–96, and the sidebar, pp. 91–92.

2 The terms “alcoholic patient” and “alcoholism” as used in this article are summary terms for the diagnoses of alcohol abuse and alcohol dependence as defined variously by the studies cited.

3 For a person weighing 150 pounds, this dose would be roughly equivalent to 17 drinks.
